# New Method of Covering the Taste of Cod Liver Oil

**Published:** 1879-01

**Authors:** 


New Method of Covering the Taste of Cod-Liver Oil.—Dr. Ponteres mixes a tablespoonful of cod-liver oil with the yellow of an egg, and when they are thoroughly combined adds to them a few drops of spirits of mint and a half a glass of sugar-water. In this way he obtains a sort of mulled egg, which differs very little from ordinary mulled egg, and which presents neither the taste nor the odor characteristic of cod-liver oil. It can consequently be taken without repugnance by the most fastidious patients.—*Union Med.*

